# Poly-G/poly-C tracts in the genomes of *Caenorhabditis*

**DOI:** 10.1186/1471-2164-8-403

**Published:** 2007-11-07

**Authors:** Yang Zhao, Nigel J O'Neil, Ann M Rose

**Affiliations:** 1 Department of Medical Genetics, University of British Columbia, Life Sciences Centre, Room 1364 – 2350 Health Sciences Mall, Vancouver, BC, V6T1Z3, Canada

## Abstract

**Background:**

In the genome of *Caenorhabditis elegans*, homopolymeric poly-G/poly-C tracts (G/C tracts) exist at high frequency and are maintained by the activity of the DOG-1 protein. The frequency and distribution of G/C tracts in the genomes of *C. elegans *and the related nematode, *C. briggsae *were analyzed to investigate possible biological roles for G/C tracts.

**Results:**

In *C. elegans*, G/C tracts are distributed along every chromosome in a non-random pattern. Most G/C tracts are within introns or are close to genes. Analysis of SAGE data showed that G/C tracts correlate with the levels of regional gene expression in *C. elegans*. G/C tracts are over-represented and dispersed across all chromosomes in another *Caenorhabditis *species, *C. briggsae*. However, the positions and distribution of G/C tracts in *C. briggsae *differ from those in *C. elegans*. Furthermore, the *C. briggsae dog-1 *ortholog CBG19723 can rescue the mutator phenotype of *C. elegans dog-1 *mutants.

**Conclusion:**

The abundance and genomic distribution of G/C tracts in *C. elegans*, the effect of G/C tracts on regional transcription levels, and the lack of positional conservation of G/C tracts in *C. briggsae *suggest a role for G/C tracts in chromatin structure but not in the transcriptional regulation of specific genes.

## Background

Non-protein encoding DNA performs a variety of important biological functions (reviewed in [1]). However, many of the functions of non-coding DNA are poorly understood. One such non-coding DNA element is guanine-rich DNA, which has been characterized in several functional domains: the telomeres, the ribosomal DNA and, in mammals, the immunoglobulin heavy-chain switch regions [2]. These G-rich DNA elements all have stretches of consecutive guanines and have the capacity to form secondary structures such as G-quadruplex or G4 DNA by Hoogsteen bonding [3]. G4 DNA has been proposed to have multiple biological functions *in vivo *including the regulation of gene expression and chromosome dynamics [2]. It has been hypothesised that the G4 conformation in RNA transcripts derived from G-rich DNA may be targets of transcriptional regulation based on the findings that many factors associated with RNA processing, including hnRNP D, hnRNP A1, and nucleolin, bind G4 DNA through their conserved RNA-recognition motif and RNA-binding domain [4-6]. More direct evidence for G-rich DNA affecting the transcription regulation was the finding that the G-rich DNA segment in the human c-*myc *promoter, which could form G4 DNA *in vitro*, functions as a repressor element [7]. Many studies have also suggested a possible role for G-rich motifs in chromosome dynamics. Sen and Gilbert originally proposed that the G-rich telomeres and internal chromosomal motifs in homologous chromosomes may participate in pairing during meiotic prophase, based on their observation that G-rich motifs in DNA can form parallel four-stranded G4 DNA [8]. Many proteins involved in chromosome synapsis and recombination were also found to interact with the G-rich DNA motifs [9-12]. Furthermore, the synaptonemal complex lateral element component Hop1 plays key roles in meiotic chromosome pairing, promoting synapsis of double-stranded DNA helices *in vitro *via the formation of G4 DNA [13,14]. In addition to the *in vitro *evidence, G4 DNA structures formed by G-rich DNA in the immunoglobulin switch region were shown to be present *in vivo*, and are proposed to play a role in the immunoglobulin class switch recombination [4,15]. These findings support the possibility of G-rich DNA being involved in chromosome dynamics.

The probability of one stretch of 18 guanines occurring by chance in the 100 Mb AT-rich *C. elegans *genome (GC content 36%) [16] is approximately 1/6 to the 18^th ^power, 1 in 100 trillion. In the *Caenorhabditis elegans *genome there are approximately 400 such homopolymeric poly-G/poly-C tracts (G/C tracts). Thus, these tracts are greatly over-represented in the genome. A study by Denver and colleagues on homopolymeric nucleotide (HP) runs in *C. elegans *reported that the observed number of the G/C tracts is much greater than expected. While the number of A/T tracts declines steadily with the increase of the length as expected, G/C tracts do not display that trend [17]. Furthermore, retention of the tracts was found to be dependent on enzymatic activity as disruption of DOG-1, a protein with similarity to the human FANCJ helicase, caused deletions that initiated in G/C tracts with no less than 18 guanines [18]. Cheung et al. proposed that DOG-1 may prevent deletions of G/C tracts by unwinding G-rich secondary structures arising during lagging strand DNA synthesis [18]. Youds et al. demonstrated involvement of the homologous recombination repair pathway in the prevention of G/C tract deletions in the *dog-1 *mutant [19]. Based on the observations that G/C tracts are over-represented in the *C. elegans *genome and protected by enzymatic activity, it seems unlikely that the occurrence and maintenance of G/C tracts are by chance.

In this study, we characterize the frequency and distribution of G/C tracts in two species of nematode, *C. elegans *and *C. briggsae *and explore possible biological roles of these tracts in these two organisms.

## Results

### G/C tracts are over-represented in the *C. elegans *genome

**Figure 1:**
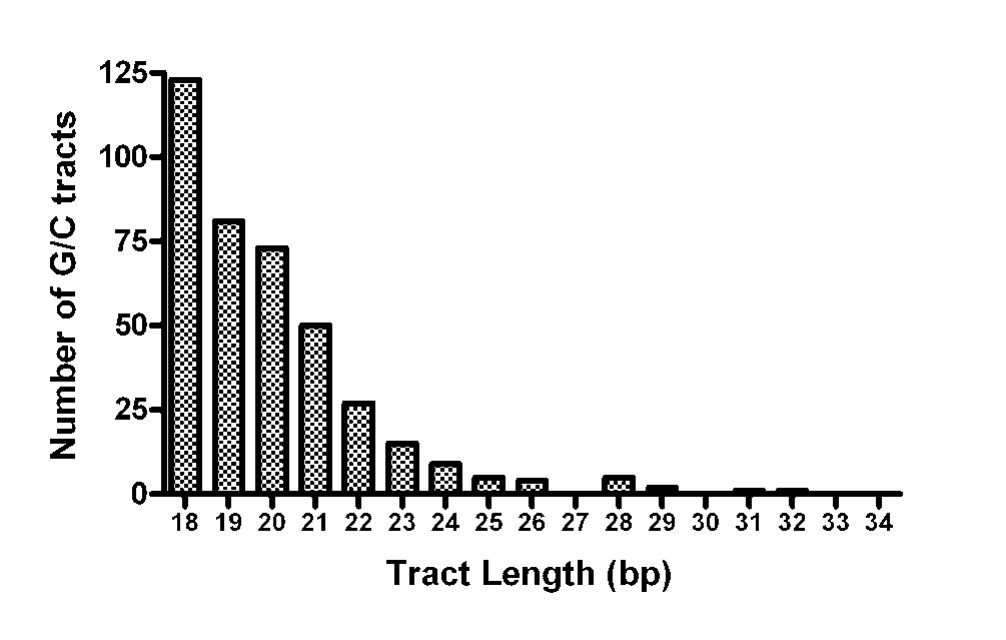
Length distribution of G/C tracts in *C. elegans *genome.

Although statistically no G/C tracts containing 18 or more consecutive Gs are expected in the 100 Mbp *C. elegans *genome, 396 G/C tracts were found. They are over-represented in the *C. elegans *genome, especially compared to the human (200 in 3.3 Bbp) and yeast (1 in 12 Mbp) genomes. These G/C tracts range in size from 18 to 32 base pairs and the frequency decreases with increased length (Figure 1). The tracts are distributed throughout the genome along all six chromosomes. The five autosomes have approximately 50 to 70 tracts each, while the X chromosome has more than 100 tracts. The density of G/C tracts on LGX is 6.1 per Mbps compared to 2.7 per Mbps of LGV (longest chromosome in *C. elegans*) (Table 1).

**Table 1:** Genomic location of G/C tracts in *C. elegans*

				Location		
				
LG	Phys. Length (Mb)	G/C tracts	Density (per Mb)	Left	Mid	Right
I	15	69	4.6	29 (42%)	11 (16%)	29 (42%)
II	15	52	3.5	27 (52%)	6 (11%)	19 (37%)
III	14	58	4.1	22 (38%)	14 (24%)	22 (38%)
IV	17	51	3.0	32 (63%)	7 (14%)	12 (23%)
V	21	57	2.7	28 (49%)	5 (9%)	24 (42%)
X^a^	18	109	6.1	45 (41%)	19 (18%)	45 (41%)

a: There is no gene cluster center and arm differentiation on the X chromosome [24]. In this study, the LGX was divided into three parts based on its physical length.

### G/C tracts are conserved in *C. elegans *Hawaiian (CB4856) isolate

Given that G/C tracts are unstable in the *C. elegans dog-1 *mutant, we wanted to test if G/C tracts were conserved in other wild type isolates of *C. elegans *or whether G/C tracts are inherently unstable. Recent studies using array comparative genomic hybridization (aCGH) demonstrated that there is approximately 2% gene content variance between the Hawaiian (CB4856) and Bristol (N2) isolates, and that these differences are primarily deletions in the CB4856 Hawaiian strain [20]. Furthermore there are a large number of single nucleotide polymorphisms (SNPs) between the two intraspecific isolates, 10,711 for 7.3% covered sequence [21]. We assayed the conservation of G/C tracts in the CB4856 Hawaiian strain by PCR and sequencing. One random chosen G/C tract on each chromosome was analyzed and the sequencing results showed that the presence, length and orientation of G/C tracts were conserved in the Hawaiian strain. This result demonstrated the preservation of the tested G/C tracts in these two wild type isolates of *C. elegans*.

### G/C tracts are also over-represented in *Caenorhabditis briggsae*

To determine if the location and orientation of G/C tracts are conserved in closely related nematode species, we investigated the G/C tract conservation in *C. briggsae*, a closely related species of *C. elegans*. In total, 216 G/C tracts in size from 18 to 25 base pairs were found in the *C. briggsae *genome based on the sequence release cb25.agp8 [22,23], of which 46 G/C tracts are intragenic and 170 G/C tracts are intergenic. Although there are fewer G/C tracts in the *C. briggsae *genome compared to the *C. elegans *genome, the tracts are still over-represented as the *C. briggsae *genome is similar to that of *C. elegans *in size and GC content [22].

We found that the positions of G/C tracts are not well conserved between two species. The assay on the conservation of the G/C tracts was performed using the following procedure: 1) find the gene (for intragenic G/C tracts) or the nearest gene to a G/C tract (for intergenic G/C tracts) in *C. briggsae *genome; 2) identify the corresponding *C. elegans *ortholog; 3) screen for G/C tracts in the gene or surrounding sequence. Three intragenic G/C tracts are located within the same genes in two species: a 19 bp tract in *C. elegans *gene *vab-10 *and an 18 bp tract in its *C. briggsae *ortholog CBG15813; a 23 bp tract in *hmr-1 *and an 18 bp tract in CBG07964; and a 22 bp tract in H10D18.5 with its counterpart a 20 bp tract in CBG01202. However, only the tracts in *vab-10 *and CBG15813 could be considered conserved when the facts such as tract orientation and position specificity were taken into account (Table 2). For intergenic G/C tracts, 14 G/C tracts were found to be located in similar locations in *C. elegans *and *C. briggsae*. However, using a strict criterion (presence, orientation, and position), only 4 G/C tracts could be classified as conserved (Table 2).

**Table 2:** Conserved G/C tracts in *C. briggsae *and *C. elegans*

	*C. briggsae *gene	*C. elegans *ortholog	*C. elegans *chromosome
Intragenic G/C tracts	***CBG15813***	***vab-10***	***I***
	CBG01202	H10D18.5	V
	CBG07964	*hmr-1*	I
			
Intergenic G/C tracts^#^	***CBG05578***	***dnj-25***	***V***
	***CBG07642***	***C18B12.2***	***X***
	***CBG13811***	***Y65A5A.1***	***IV***
	***CBG14237***	***ckc-1***	***X***
	CBG03680	ZK430.8	II
	CBG04107	F32B5.6	I
	CBG04376	F32B4.5	I
	CBG06557	F59D6.6	V
	CBG07688	C33A11.1	X
	CBG09160*	ceh-13*	III
	CBG15039	C09G12.1	IV
	CBG15729	H10E21.2	III
	CBG20844	*gur-4*	II

G/C tracts considered conserved between the two species under strict criterion were highlighted with bold font. ^# ^Intergenic G/C tracts were defined by closest gene in this table. *There are two G/C tracts in both CBG09160 of *C. briggsae *and *ceh-13 *of *C. elegans*.

The fact that the *C. briggsae *genome also possesses abundant G/C tracts but fewer than *C. elegans *led us to question whether G/C tracts are protected by a *C. briggsae *DOG-1 ortholog in a manner similar to *C. elegans*. Based on the results of amino acid reciprocal best blast hits, there is a predicted ortholog of *dog-1 *in *C. briggsae *named CBG19723. RT-PCR showed that it was transcriptionally active, producing an mRNA of 2892 base pairs. We sequenced the *C. briggsae dog-1 *cDNA and found that there was an additional exon that was not included in the predicted *C. briggsae *gene model (Wormbase cb25.agp8 [22,23]). The corrected gene model showed 71% amino acid identity and 87% protein similarity with its *C. elegans *ortholog.

Since there were no mutant alleles of CBG19723 available we tested whether the *C. briggsae *DOG-1 ortholog had a role in maintaining G/C tracts. We introduced the full length CBG19723 gene with its promoter region into the *dog-1*(*gk10*) knockout mutant to see if the *C. briggsae *DOG-1 ortholog could rescue the G/C tract deletion phenotype. Deletions were observed in the *vab-1 *G/C tract in 12/101 (11.9% CI: 6.62–20.1%) *dog-1(gk10) *animals. Deletions of the same site were observed in only 2/136 (1.47% CI: 0.26–4.92%) *dog-1(gk10); hEx264 *transgenic animals, significantly lower than non-transgenic animals (t-test, P < 0.001). This result clearly demonstrates that the *C. briggsae dog-1 *ortholog CBG19723 can protect the integrity of G/C tracts in *C. elegans*, rescuing the G/C tract deletion phenotype of the *dog-1 *mutant. Therefore, CBG19723 could be protecting the 216 G/C tracts in *C. briggsae *just as DOG-1 does in *C. elegans*. Although the positions of most G/C tracts are not conserved, the G/C tracts are protected by the *C. briggsae *DOG-1 ortholog.

### G/C tracts are distributed non-randomly on *C. elegans *chromosomes

**Figure 2:**
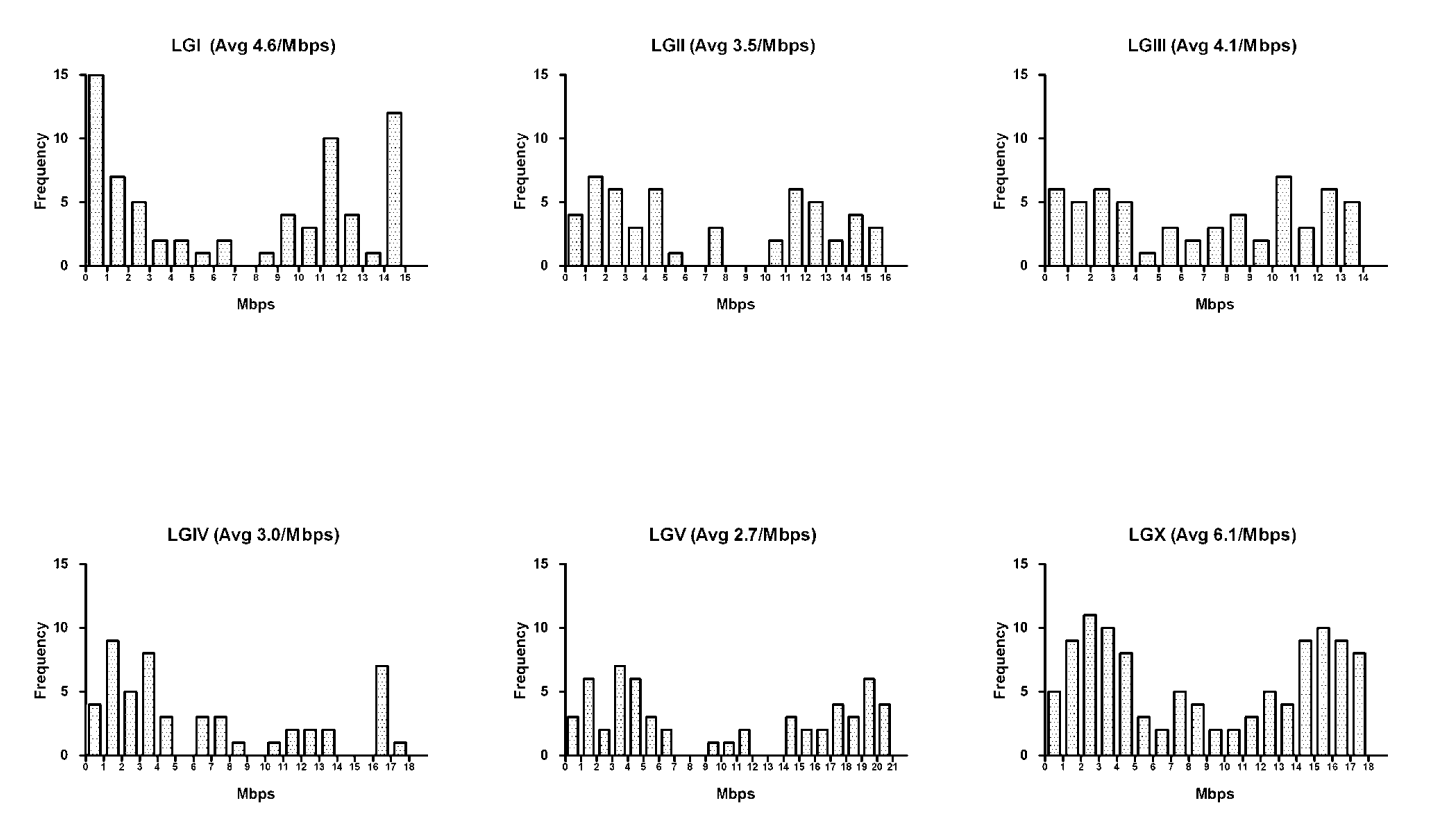
Distribution of G/C tracts in every mega-base pair block on each chromosome of *C. elegans*. In each graph, X axis represents the length of the chromosome that was divided by million base pairs, while Y axis is the frequency of G/C tracts.

**Figure 3:**
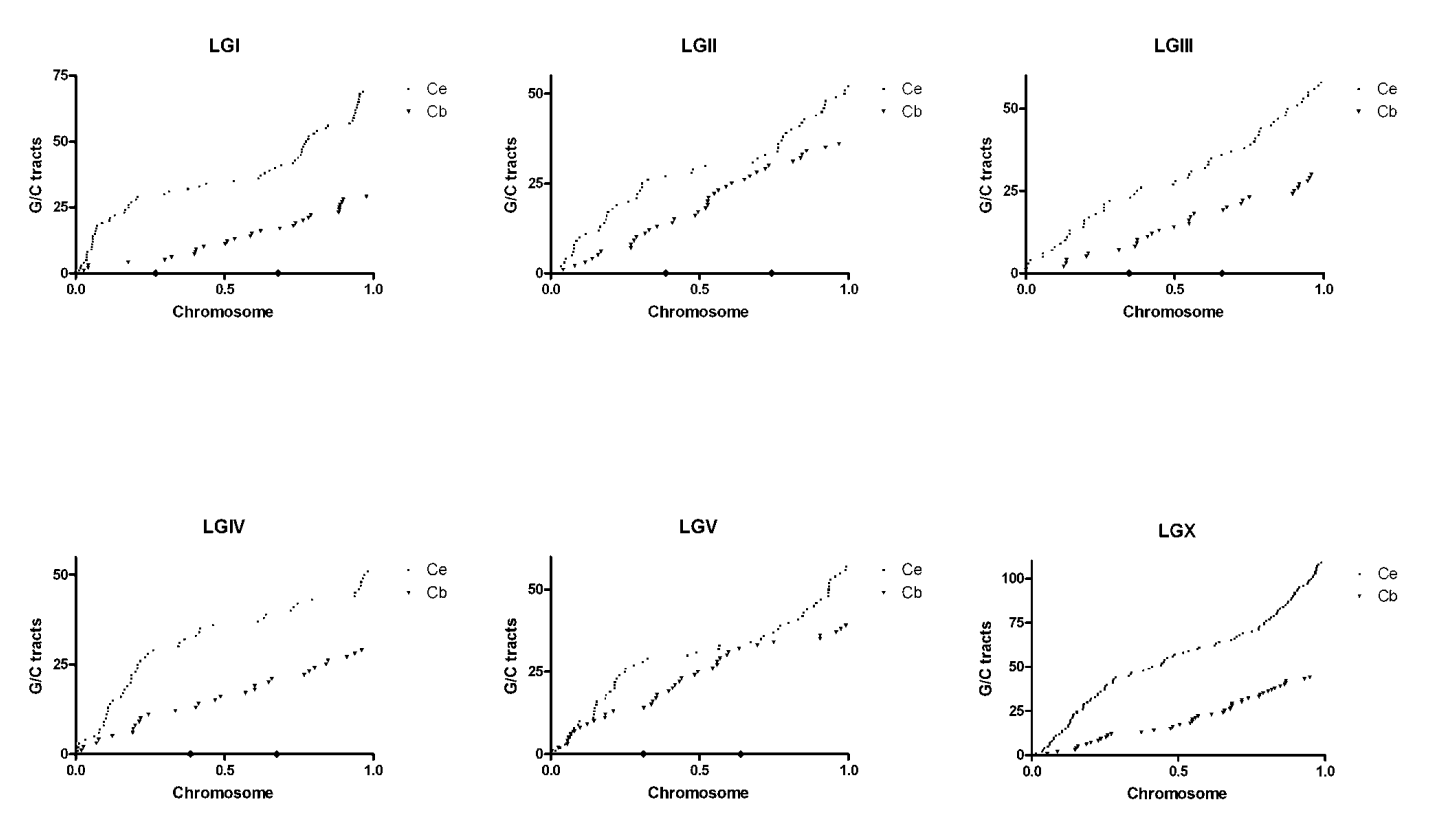
Distribution of G/C tracts along each chromosome of *C. elegans *and C. briggsae*. X axis in each graph represents one chromosome whose length was normalized to 1 and Y axis is the number of G/C tracts. Each G/C tract from the left end to the right end of one chromosome was numbered sequentially. The diamond spots on the X axis marked the edge of the genetically defined central gene cluster [24]. * Positions of G/C tracts in *C. briggsae *were predicted by the method described in text.

Although the GC content in the *C. elegans *genome is similar on all the chromosomes [16], the G/C tracts are not distributed uniformly across the chromosomes. *C. elegans *autosomes can be divided into three genetically defined compartments of the left arm (L), the central gene cluster region (C), and the right arm (R) [24]. We found that more G/C tracts are located in the chromosome arms than in the central regions (Table 1, Figure 2 and 3). While this pattern is fairly subtle on LGIII, it is more obvious on the other chromosomes especially on LGV where only 9% of G/C tracts are located in the central region while 91% reside on the arms of LGV (Table 1, Figure 2 and 3). This non-random distribution of G/C tracts on the autosomes correlates with both the meiotic cross-over distribution [24] and negatively with gene density [16,22,25]. An enrichment of G/C tracts on the chromosome arms of the X chromosome was observed even though the X chromosome does not have a central gene cluster or a meiotic cross-over pattern. The distributions of the intragenic and intergenic G/C tracts do not differ from the overall distribution pattern with more and longer G/C tracts on the arms. Nor is there any distinct pattern with regard to the orientation of G/C tracts on either DNA strand.

### G/C tracts are distributed uniformly in *C. briggsae *genome

In order to graphically demonstrate the distribution of G/C tracts in the genome, a "Marey Map" [26] approach was used. As shown in Figure 3, abundant G/C tracts in a given region result in a steeper slope than areas that lack of G/C tracts. When the distribution of G/C tracts in *C. elegans *was represented in this way, the plot resulted in "S" curves, subtle on LGIII but more obvious on the other chromosomes (Figure 3), indicating the abundance of G/C tracts on chromosome arms compared to the clusters. The curves on our map were similar to those on the genetic/physical Marey map reported by Barnes *et al*. [24], which reflected the increased meiotic crossing-over in the arm regions compared to the crossing-over in the central gene clusters.

**Figure 4:**
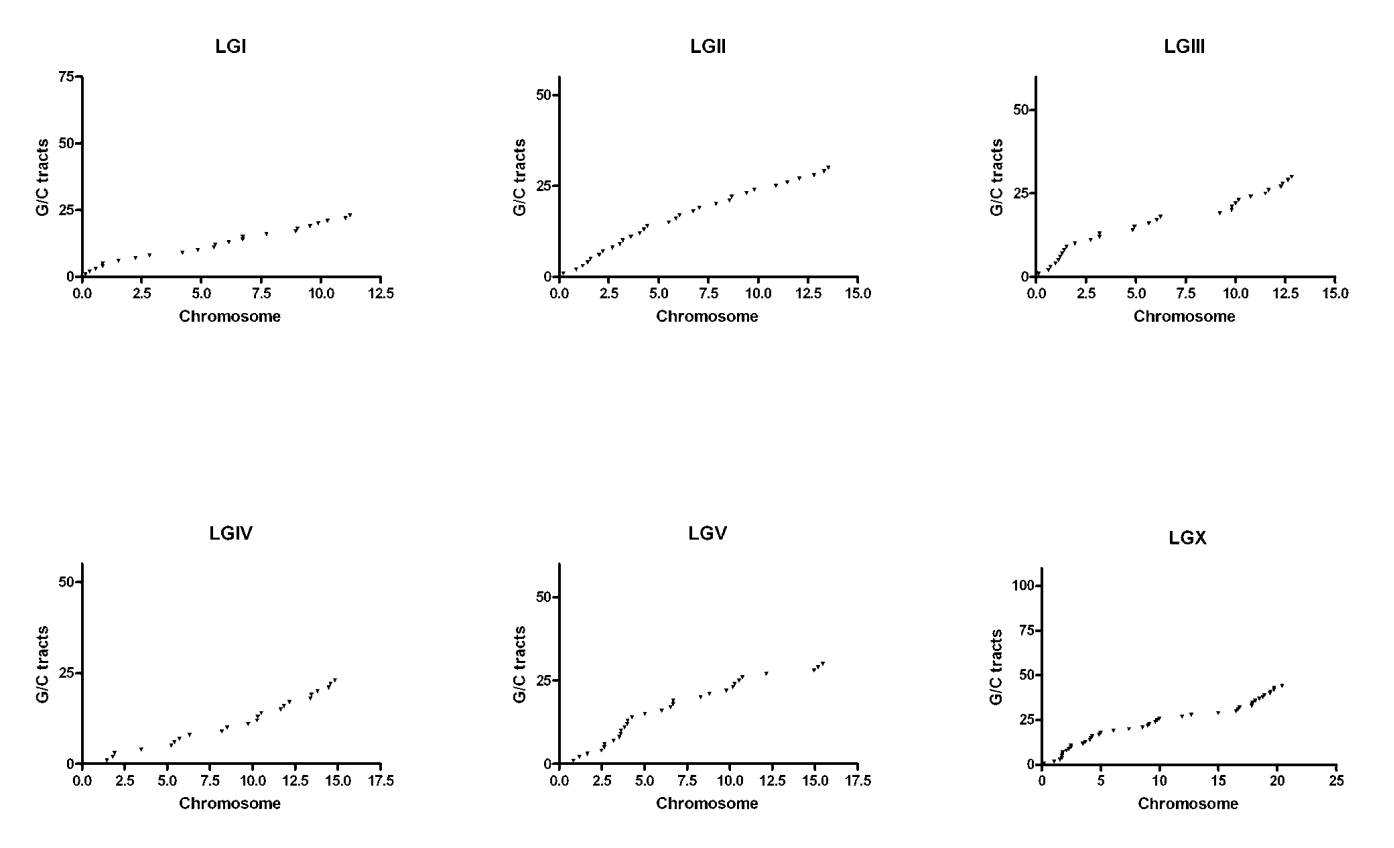
Distribution of G/C tracts along each chromosome of *C. briggsae *based on genome assembly CB3. X axis of each graph represents the physical length of each chromosome and Y axis is the ordinal number. Each G/C tract from the left end to the right end of one chromosome was numbered sequentially.

Based on the knowledge that the two genomes exhibit extensive colinearity [22,27,28], we used the genomic positions of the *C. elegans *orthologs of the G/C tracts' associated genes in *C. briggsae *to create a predicted genomic distribution map of G/C tracts in *C. briggsae*. In the *C. briggsae *plots (Figure 3), G/C tracts are also dispersed across every chromosome in the *C. briggsae *genome. Because there are fewer G/C tracts on each chromosome, the curves are beneath their *C. elegans *counterparts. The slope of the *C. briggsae *line illustrates that the G/C tracts are distributed evenly across the chromosomes. Both the analysis presented here (Figure 3) and a more recent analysis based on the chromosome-based assembly of *C. briggsae *genome (Wormbase CB3 [23,28]) result in a similar pattern (Figure 4). Thus, although there is no specific patterning to the position or the orientation of the tracts, the number and dispersed location of them in these two species is suggestive of a biological role.

### G/C tracts mostly occur between genes or in introns

Although 27% of *C. elegans *genome is in predicted exons [16], only 4 out of 396 G/C tracts (1%) were in gene exons (Y48G1BM.7, F49B2.3, Y105E8A.26, and F43b10.1). Consecutive guanines or cytosines would encode strings of glycines or prolines, an unlikely sequence combination. The four genes containing G/C tract sequences are located near the ends of the chromosome arms. The predicted gene products are not currently assigned to any KOG (Conserved Orthologous Groups) category [29,30] and as there is no EST or SAGE data, it is possible that they are not expressed. It is possible that these genes were predicted incorrectly and that the G/C tracts are not in coding regions. For the remaining 392 G/C tracts, 127 of them were found to be in non-coding regions of genes (6 in UTRs and 121 in introns), and the other 265 G/C tracts are located between genes.

The genes containing tracts vary in length, from less than 500 bp to more than 50 kb, with an average length of 10.6 kb, much longer than the average gene size for the whole genome (2.5 kb) [16]. This was true for all chromosomal regions: autosome central, 10.0 kb; autosome arm, 11.2 kb; and X chromosome, 9.0 kb. The location of the tracts within introns was unbiased, but not the orientation. Almost twice as many intragenic G tracts are located on non-coding strands as on coding strands (84 vs. 47). This preference might be due to catastrophic problems caused by higher structures formed by G/C tracts during the transcription on the non-coding strand. The number of intragenic G/C tracts on each chromosome is very similar. Thus, the high number of G/C tracts on the X chromosome is largely due to a greater number of tracts between genes on that chromosome (Table 3).

**Table 3:** Positions of G/C tracts in *C. elegans*

LG	Intergenic	5' UTR	Intron	Exon	3'UTR
I	38	0	28	3	0
II	33	0	18	0	1
III	35	0	22	0	1
IV	36	1	13	0	1
V	38	0	19	0	0
X	85	0	21	1	2

Eddy and colleagues previously reported that G-rich DNA motifs with potential to form G4 DNA are highly represented in proto-oncogenes in human genome [31]. This result prompted us to investigate whether or not the G/C tract bearing genes in *C. elegans *are functionally related. Sixty-nine percent of the genes containing G/C tracts could be assigned to a KOG classification (Table 4). The most abundant category "poorly characterized genes" (31 genes) combined with the number of unassigned genes (41 genes) account for more than half of the total G/C tract bearing genes, indicating that many G/C tract bearing genes are not well understood. "Signal transduction mechanisms" and "Transcription" are the two most abundant categories with specified functions, and contain 23 and 11 genes respectively. However, they are not statistically over-represented based on the *C. elegans *whole genome KOG classification [30] (Chi-Square test, P > 0.05). Overall, there is no evidence that the G/C tract bearing genes are functionally related.

**Table 4:** KOG classification of intragenic G/C tracts bearing genes

KOG classifications	Genes	Categories	Genes
CELLULAR PROCESSES AND SIGNALING	36	Posttranslational modification, protein turnover, chaperones	5
		Signal transduction mechanisms	23
		Defense mechanisms	1
		Extracellular structures	3
		Cytoskeleton	4
INFORMATION STORAGE AND PROCESSING	15	RNA processing and modification	2
		Chromatin structure and dynamics	1
		Translation, ribosomal structure and biogenesis	1
		Transcription	11
METABOLISM	9	Cell cycle control, cell division, chromosome	2
		Amino acid transport and metabolism	2
		Carbohydrate transport and metabolism	1
		Inorganic ion transport and metabolism	3
		Secondary metabolites biosynthesis, transport and catabolism	1
POORLY CHARACTERIZED	31	General function prediction only	18
		Function unknown	13
Unassigned	41		

*The G/C tract in T06A10 on LGIV was located in two overlapping genes T06A10.4 and *mel-46*, both genes were analyzed in this study.

The average distance from an intergenic G/C tract to the nearest gene is 1.9 kb. Although some of the 265 intergenic G/C tracts are located as far as nearly 10 kb from the nearest gene, most intergenic G/C tracts are found to be close to genes. Most intergenic G/C tracts (197 out of 265, 74%) are found to be within 3 kb of the nearest gene and almost one third of intergenic G/C tracts (85 out of 265, 32%) are within 500 bp of the nearest gene. Thirteen G/C tracts were found to reside between two genes that are closer than 1 kb. Interestingly, although it is known that the gene density in the central gene cluster regions on autosomes is higher than autosome arms [16,24], the average distance of the intergenic G/C tracts from a gene in autosome central gene cluster regions is actually slightly larger than that on the arms (1.96 kb vs. 1.83 kb).

### G/C tracts are associated with the levels of regional gene expression

Both introns and the flanking regions of genes are known to affect gene expression. We thus suspected that the abundant G/C tracts in these areas are associated with the transcriptional regulation of corresponding genes. We investigated the relationship between the position of the G/C tract and the level of transcription using *C. elegans *SAGE (Serial Analysis of Gene Expression) data [32]. SAGE data for genes containing intragenic G/C tracts and for various distances away from a G/C tract was assembled and collated (Table 5). Only the specific tags assigned to genes (A "specific" tag is defined as a tag that uniquely matches to a single gene or that can be resolved to a single gene by taking the lowest position match [32]) were used. Genes with a G/C tract within an intron (intragenic tracts) had on average 2.05 SAGE tags (2.21 tags if on the non-coding strand and 1.77 tags if on the coding strand). Similarly, genes within 500 bp of a G/C tract had 1.92 tags. Genes 500–1500 bp away from a G/C tract had on average 1.19 tags and those 1.5–3 kb had 1.50 tags. Genes further away, 3–5 kb, exhibited higher levels of gene expression. The average of 4.48 tags per gene differed significantly from the averages for genes closer to tracts (P value of t-test to intra G/C genes: 0.039; to genes within 500 bp: 0.053; to genes in 500 bp-1.5 kb: 0.008; to genes in 1.5–3 kb: 0.017). For genes 5 kb away from G/C tracts the average SAGE tag number is 3.00. Thus, genes located distantly from G/C tracts have higher levels of transcription than genes close to G/C tracts, which may reflect a relationship to chromatin domains. While genes on the autosome arms in *C. elegans *tend to be poorly expressed [16], average number of SAGE tags of genes associated with G/C tracts located on autosome arms does not significantly differ from that of those in the central gene clusters (2.97 vs 2.49 respectively, t-test P > 0.5). There is no significant difference in SAGE tag number whether the G tract is on the coding strand or not, and whether the G/C tract is upstream (5' end) or downstream (3' end). Spearman correlation coefficient analysis revealed correlation between the SAGE tag numbers and gene distance from a G/C tract for genes that are no more than 3 kb away from a G/C tract (P < 0.005). This correlation was not observed on genes further away.

**Table 5:** Average SAGE tags of genes associated with nearby G/C tracts

Position	G/C bearing genes N = 132*	< 500 bp N = 104	0.5–1.5 kb N = 110	1.5–3 kb N = 127	3–5 kb N = 136	5–10 kb N = 179
SAGE tags	2.05	1.92	1.19	1.50	4.48	3.00

*The G/C tract in T06A10 on LGIV was located in two overlapping genes T06A10.4 and *mel-46*, both genes were analyzed in this study.

## Discussion

Both the *C. elegans *and *C. briggsae *genomes contain many more G/C tracts than expected as not even one tract greater than 18 bps is expected to be found by chance in a genome of 100 Mb of DNA. Most G/C tracts are located in intergenic or intronic areas. Genes containing tracts are much larger than average and more G/C tracts are located on the gene-poor chromosomal arms. Thus, the tracts are located in regions with a low density of coding DNA. However, the fact that both the *C. elegans *and *C. briggsae *genomes contain a large number of these unusual homopolymeric tracts distributed across each chromosome and the existence of a conserved protein, DOG-1, that prevents the deletion of G/C tracts support the possibility that G/C tracts have biological significance.

Initially it would appear that the X chromosome in *C. elegans *is significantly different than the autosomes with regard to G/C tracts distribution. The X chromosome contains almost twice as many G/C tracts as the autosomes whereas both inverted and tandem repetitive sequences are more frequent on the autosomes than on the X chromosome [16]. For example, CeRep11, with 711 copies distributed over the autosomes, has only one copy located on the X chromosome [16]. Although the X chromosome has many more G/C tracts than the autosomes, it has approximately the same number of intragenic G/C tracts (24/109) compared to the autosomes (Avg. 21.4 each), which means the large number of G/C tracts on the X are due to a larger number of intergenic G/C tracts (85 vs. 36 of autosomes) (Table 3). Furthermore, the overall gene density of the X chromosome is lower than that of the autosomes [16]. Taken together with regard to the distribution of G/C tracts on X chromosome, it would appear that the X chromosome is organized like an autosomal arm. More G/C tracts were found to be located on the arms of the X chromosome even though the X chromosome does not have a meiotically defined central gene cluster, suggesting that there is no correlation between the meiotic pattern and G/C tracts. Recent study on *C. briggsae *genetic mapping using SNPs showed that *C. briggsae *has a similar meiotic pattern as *C. elegans *[28]. This finding, along with our observation of the uniform distribution of G/C tracts in *C. briggsae*, also suggests that G/C tracts are not correlated with meiotic recombination.

We examined in *C. elegans *the distribution of the tracts in the context of the level of gene expression. Based on SAGE analysis, we observed a variation of transcription levels that correlates with distance from the G/C tract. The average number of SAGE tags associated with G/C tract flanking genes is significantly lower than the number of SAGE tags associated with genes further from G/C tracts. This effect is similar to the suppression of human c-*myc *gene by its G-rich promoter [7]. Although no evidence for direct regulation by G/C tracts on specific genes was found in *C. elegans*, our observation showed that there is a correlation with the presence of a G/C tract and the level of expression. If G/C tracts played important gene specific cis-regulatory functions they should be conserved in *C. briggsae *but few are. Thus, we believe G/C tracts are unlikely to be regulating transcription in a gene specific manner. On the other hand, previous studies on regulatory elements in *C. elegans *muscle genes showed that regulatory elements are also highly represented in *C. briggsae *muscle genes but the conservation of individual sites is weak [33]. One of their interpretations was that a specific site in *C. elegans *could disappear over evolutionary time and reappear at a different position and retain its regulatory activity. Similarly, G/C tracts in *C. elegans*, although not conserved in position in *C. briggsae*, could be affecting regional gene expression. This may be due to changes in the local chromatin environment, perhaps as a result of the G/C tract that affects gene transcription, or that these chromatin environments may be more conducive to the generation or maintenance of G/C tracts.

The capability of G-rich secondary DNA structures to interact with each other using non Watson-Crick base pairing makes it an excellent candidate to coordinate chromosome dynamics such as pairing during meiosis. Sen and Gilbert originally proposed that G4 DNA might facilitate the pairing of homologous chromosomes without the need for testing homology at the sequence level [8]. There are several lines of evidence suggesting that the G/C tracts in *C. elegans *might have a role in meiotic chromosome pairing. Many pairing or recombination related components can interact with G-rich DNA secondary structures [9-14]. For example, the mismatch repair protein MutSα binds G4 DNA and promotes synapsis and recombination in the mammalian immunoglobulin switch regions [34] and in yeast, the meiotic synapsis protein Hop1 can promote the formation of G4 DNA and the synapsis of double-stranded DNA helices through the generation of G4 DNA [12-14]. The distribution of G/C tracts across the chromosomes in two species is compatible with a role for the tracts in chromosome alignment for pairing.

In many organisms, homolog pairing is achieved by double-stranded DNA breaks and Rad51-mediated strand invasion. Subsequent strand invasion can then align and pair homologous chromosomes before the formation of the synaptonemal complex. In *C. elegans *and *D. melanogaster*, this is not the case as alignment and synapsis occurs without the generation of DSBs (reviewed by Joyce and McKim [35]). Although the specific mechanisms that drive homologous chromosome pairing while preventing the pairing of non-homologous chromosomes are unknown, DNA domains in *C. elegans *chromosomes have been proposed to play key roles in homolog pairing. Genetic analysis of *C. elegans *chromosome rearrangements identified cis-acting regions for pairing [36] which occur at one 'end' of each *C. elegans *chromosome which were called the homolog recognition region (HRR) [37]. Recent reports showed that specific C2H2 zinc-finger proteins bind to these regions and mediate the chromosome synapsis [38,39], however, they are not essential for homolog pairing [39,40]. Once synapsis initiates, it is likely that sequences distributed along the chromosomes promote proper alignment. In the case of large deletions or insertions that disrupt alignment, pairing for recombination can reinitiate, thus, there must be some mechanism by which homologous chromosomes can be brought into register along the length of the chromosomes without stand invasion [41,42]. Abundant G/C tracts distributed along the chromosomes with the ability to generate secondary G-rich structures that can pair with other G-rich structures on the homologs could function to align homologous chromosomes. The observation that yeast Hop1 protein is able to promote synapsis of double-stranded DNA helices by the formation of G4 DNA raised the possibility that Hop1 homologs in *C. elegans *(*him-3 *and the *him-3 *paralogs) might have similar capabilities. This speculation is supported by the fact that mutants of *him-3 *and *him-3 *paralogs *htp-1, htp-2 *were reported to all have chromosome pairing defects [43-45]. It will be interesting to investigate whether *him-3 *or the *him-3 *paralogs can interact with G/C tracts.

## Conclusion

In this article, we reported our analysis on the G/C tracts in *C. elegans *genome and their conservation in another *Caenorhabditis *species, *C. briggsae*. Overrepresented G/C tracts are dispersed throughout the genomes of these two species, but the specific positions and the overall distribution patterns do not appear to be conserved. Along with the finding that G/C tracts are correlated with the levels of regional gene expression in *C. elegans*, we proposed that G/C tracts have possible biological roles in chromosome dynamics and/or gene expression regulation.

## Methods

### Data collection and computational analysis

G/C tracts data including the length, orientation, and position as well as the other related genomic information in the *C. elegans *and *C. briggsae *genome, were obtained from the genomic DNA sequence database available on Wormbase (*C. elegans *release WS165, *C. briggsae *release CB25) [23]. SAGE data were obtained from the Genome BC *C. elegans *Gene Expression Consortium [32,46].

### Nematode strains

The strains used include: N2 Bristol strain (wild-type), CB4856 Hawaiian strain (wild-type), VC13 *dog-1 *(*gk10*), and AF16 *C. briggsae *strain. Strains denoted with the *h *prefix arose in the A.M. Rose lab. All strains were maintained as previously described [47].

### CBG19723 cloning and microinjection

The full-length genomic DNA with the 194 bp promoter region of CBG19723, ortholog of *dog-1 *in *C. briggsae*, was amplified by the Finnzymes Phusion High-Fidelity Polymerase using primers 5'-atactcgagcgaaaattccagaaaatttggc-3' and 5'-ataactagtcatgcgtcctcctgctccttctt-3'. The PCR fragment was then cloned into the *Xho*1 and *Spe*1 sites of pBluescriptII/KS(+) vector. This plasmid (named as pYZ1) (12 ng/ul) and the pRF4 *rol-6(su1006) *(60 ng/ul) marker plasmid were microinjected into the germ lines of N2 adults to form the transgenic array *hEx264*. *dog-1(gk10)/dog-1(gk10)*; *hEx264 *strain was then made by crossing the *hEx264 *to *dog-1(gk10) *animals. Successful transgenic *dog-1(gk10) *animals were tested for rescue of *dog-1 *mutant phenotype using the deletion frequency of the G/C tract in gene *vab-1 *[19].

A full-length CBG19723 cDNA was isolated by Reverse Transcriptase-PCR (RT-PCR) of total RNA from mixed-stage *C. briggsae *cultures. The RT-PCR product was cloned in to the pGEM-T vector (Promega) and then verified by sequencing (Nucleic Acid and Protein Services, NAPS, UBC).

### Measurement of G/C tract deletion frequency

L4 stage animals of the genotype of interest were picked to fresh plates 24 hr before DNA preparation. DNA of individual worms was prepared with lysis buffer (10 mm Tris-HCl, 50 mm KCl, 2.5 mm MgCl2, 0.45%NP40, 0.45% Tween20, 0.01% gelatin, 100 mg/ml ProteinaseK) and incubated at -70°C for 10 min, at 60°C for 1 hr, and then at 95°C for 15 min.

G/C tract deletion within the *vab-1 *gene on chromosome II was measured by PCR as described [19]: G/C tract and flanking DNA were amplified in each animal by PCR using a set of nested primers. External primer sequences were 5'-cgattccaacaattggtaaatacc-3' and 5'-aatatttgctaaacctattgttgcc-3'. The external PCR program was 94°C for 4 min followed by 34 cycles of 94°C for 30 sec, 58°C for 30 sec, and 72°C for 1 min 30 sec, and a final elongation step of 72°C for 10 min. One microliter of DNA from the external reaction was used as the template for a second internal PCR. Internal primer sequences were 5'-cgacgaaaaatgcagaatttggc-3' and 5'-aggtgtgtgtgcatacctccg-3'. The internal PCR program was the same as the external program, except primers were annealed at 62°C and the extension time was 1 min. PCR products were run on 1% agarose gels and stained with SYBR Green (Molecular Probes) for nucleic acid visualization. Gels were imaged using a Gel Doc 2000 (Bio-Rad, Hercules, CA).

Other G/C tract amplification and deletion tests in this report used similar protocol but nested primers were not used.

### Verification of G/C tracts in Hawaiian strain

Six random chosen G/C tract sites (one on each chromosome) of Hawaiian strain CB4856: K09H9 (LGI, primers: 5'-ctcgaacggaaatgtcaatatgg-3' and 5'-ctgcgttactttgactatcagag-3'), M03A1 (LGII, primers: 5'-cgacgaaaaatgcagaatttggc-3' and 5'-aggtgtgtgtgcatacctccg-3'), R144 (LGIII, primers: 5'-catatggattggcatgtgaagca-3' and 5'-tcaactttgacagcatttatccga-3'), F07C6 (LGIV, primers: 5'-cacgcttatcatttcaaatgtac-3' and 5'-cgagcacaagtggcacatcgg-3'), T22H9 (LGV, primers: 5'-cccaacaactcgtatgccatc-3' and 5'-cgcgggaatatctaaattgtcta-3'), and Y9C12A (LGX, primers: 5'-cttgaagagaattccgaatgaaac-3' and 5'-ctcattgccaaactcctccac-3'), were analyzed by DNA sequencing on the PCR products amplified using primers flanking the G/C tracts. DNA preparation and PCR protocol were same as described above.

## List of abbreviations

CGH: comparative genomic hybridization;

DSB: double-stranded break;

EST: expressed sequence tag;

HRR: homolog recognition region;

KOG: conserved orthologous groups;

LG: linkage group or chromosome;

SAGE: serial analysis of gene expression;

SNP: single nucleotide polymorphisms.

## Authors' contributions

YZ carried out the molecular genetic studies, genomic data collection and analysis and drafted the manuscript. NO participated in the design of the study and helped to draft the manuscript. AR conceived of the study, and participated in its design and coordination and helped to draft the manuscript. All authors read and approved the final manuscript.
